# Rhubarb Protect Against Tubulointerstitial Fibrosis by Inhibiting TGF-β/Smad Pathway and Improving Abnormal Metabolome in Chronic Kidney Disease

**DOI:** 10.3389/fphar.2018.01029

**Published:** 2018-09-13

**Authors:** Zhi-Hao Zhang, Ming-Hua Li, Dan Liu, Hua Chen, Dan-Qian Chen, Ning-Hua Tan, Shuang-Cheng Ma, Ying-Yong Zhao

**Affiliations:** ^1^Key Laboratory of Resource Biology and Biotechnology in Western China, Ministry of Education, School of Life Sciences, Northwest University, Xi’an, China; ^2^State Key Laboratory of Natural Medicines, School of Traditional Chinese Pharmacy, China Pharmaceutical University, Nanjing, China; ^3^National Institutes for Food and Drug Control, State Food and Drug Administration, Beijing, China

**Keywords:** chronic kidney disease, rhubarb, TGF-β/Smad signaling, metabolomics, tubulointerstitial fibrosis

## Abstract

Tubulointerstitial fibrosis is the final common pathway for all kidney diseases leading to chronic kidney disease (CKD). TGF-β/Smad signaling pathway plays a key role in renal fibrosis. Previous studies have revealed that rhubarb extracts attenuated the increase of transforming growth factor-β 1 (TGF-β1) in CKD rats. To gain an in-depth insight into the mechanism of the anti-fibrotic activities of the rhubarb extracts, we investigated the influence of rhubarb extracts on TGF-β/Smad signaling pathway and the influence on metabolome in a rat model of CKD with adenine-induced chronic tubulointerstitial nephropathy. Male Sprague-Dawley rats were divided into four groups, including control, CKD, CKD + petroleum ether extract, CKD + ethyl acetate extract, and CKD + *n*-butanol extract groups. Kidneys harvested on the week three were evaluated for renal fibrosis, the expression of proteins in TGF-β/Smad signaling pathway and metabolomic study. We found rhubarb extracts suppressed TGF-β/Smad3-mediated renal fibrosis by reducing the TGF-β1, transforming growth factor-β receptor I (TGF-β RI), transforming growth factor-β receptor II (TGF-β RII), Smad2, p-Smad2, Smad3, p-Smad3, and Smad4, meanwhile increased Smad7. In addition, rhubarb extracts mitigated renal injury and dysfunction, and either fully or partially reversed the abnormalities of tissue metabolites. Thus, rebalancing the disorder of TGF-β/Smad signaling and metabolic dysfunction by treatment with rhubarb extracts may represent as an effective therapy for CKD associated with fibrosis.

## Introduction

The progression of CKD is an increasingly common condition and a considerable proportion of CKD eventually progress to end-stage renal disease (ESRD), a devastating condition that requires life-long treatments with dialysis or renal allograft transplantation. CKD shares a common appearance of glomerulosclerosis, vascular sclerosis, and tubulointerstitial fibrosis (Fogo, 2007). Among them, tubulointerstitial fibrosis is considered to be the final common pathway for all kidney diseases leading to chronic renal failure (Zeisberg and Neilson, 2010). Tubulointerstitial fibrosis is a dynamic process including four overlapping phases: infiltration of inflammatory cells, fibroblast activation and expansion from various sources, production and deposition of a large amount of ECM components, and tubular atrophy and microvascular rarefaction (Liu, 2011). Any of these pathologic features can contribute to the progression of tubulointerstitial fibrosis in its own unique way. Excessive accumulation of ECM and fibroblast is the main features of tubulointerstitial fibrosis (Zeisberg and Neilson, 2010). Process of tubulointerstitial fibrosis involves the interaction of a variety of cytokines and multiple signaling pathways, which leads to tubular atrophy, tubulointerstitial fibrosis and eventually loss of kidney function (Boor et al., 2010).

Transforming growth factor-β (TGF-β) is a key pro-fibrotic mediator in renal fibrosis, mostly by inducing ECM production and proliferation of myofibroblasts and fibroblasts, but also through immunoregulatory functions (Boor et al., 2010; Chen et al., 2018b). Numerous studies indicate that TGF-β and its downstream signaling cascades play an important role in activating cellular pathomechanisms that underlie the progression of renal diseases (August and Suthanthiran, 2003; Chen et al., 2018a; Hu et al., 2018). Among these downstream signals, Smad signaling is recognized as a major pathway of TGF-β signaling in progressive renal fibrosis. During fibrogenesis, Smad3 is extensively activated (Meng et al., 2013). However, Smad7 which is an inhibitory regulator in the TGF-β/Smad signaling pathway is down-regulated (Liu et al., 2013; Wang et al., 2018a,b). Activation of Smad3 associated with progressive degradation of Smad7, results in accumulation of myofibroblasts, overproduction of ECM, and reduction in ECM degradation in the kidney (Meng et al., 2015). Taken together, TGF-β/Smad signaling axis has a critical role in progression of renal fibrosis. Thus, rebalancing the TGF-β/Smad signaling by downregulating Smad3 and upregulating Smad7 might be an effective strategy for treatment of renal fibrosis.

Rhubarb, a well-known traditional Chinese medicine, has been widely used in China and Japan to treat CKD and reveals promising clinical prospects (Li and Wang, 2005). In addition, Yokozawa et al. (1984) reported rhubarb extract significantly decreased BUN and Scr in adenine-induced chronic renal failure rats. Zhang and el Nahas (1996) reported rhubarb extract decreased proteinuria and glomerulosclerosis in 5/6 nephrectomized rats. Our previous studies indicated that rhubarb extract improved renal function and attenuated up-regulation of pro-fibrotic proteins including TGF-β1 (Zhang et al., 2015, 2016a). However, the underlying molecular biology and biochemistry mechanisms of its nephroprotective effect in CKD remain uncertain.

Numerous studies have demonstrated UPLC-QTOF/HDMS is the most suitable platform for untargeted metabonomics due to its high analytic speed, high sensitivity, and high resolution for complex biological samples (Zhao et al., 2013a,b, 2014a,b; Zhang et al., 2013; Zhao and Lin, 2014; Chen et al., 2016b). Recently, metabolomics is widely applied to evaluation of drug efficacy and toxicity in traditional Chinese medicines (Zhao et al., 2012c; Chen et al., 2014, 2015, 2016a; Miao et al., 2016; Wang et al., 2017b). In this work, a non-targeted metabolomics approach was applied to investigate the metabolic profiling and the response to treatment with three different rhubarb extracts including PE, EA, and BU in rats with adenine-induced CKD. Additionally, we hypothesize that rhubarb extracts exert their nephroprotective effect by rebalancing disturbed TGF-β/Smad signaling pathway. To validate our hypothesis, we would focus on the changes in TGF-β/Smad signaling pathway in response to the treatment with rhubarb extracts.

## Materials and Methods

### Chemicals and Reagents

Creatinine, arachidonic acid (AA), docosahexaenoic acid and uric acid were obtained from the National Institutes for Food and Drug Control. L-valine was purchased from Amresco Company. Kynurenic acid, hypotaurine were purchased from Sigma Company or Aladdin Company. Antibodies against transforming growth factor-β 1 (TGF-β1), TGF-β RII, TGF-β RI, Smad2, Smad3, p-Smad2, p-Smad3, Smad4, Smad7, α-SMA, collagen I, fibronectin, E-cadherin, FSP1, vimentin, collagen III, and GAPDH were purchased from Santa Cruz Biotechnology, Cell Signaling Technology, or Abcam Company. Ultra purity water was prepared using a Milli-Q water purification system (Billerica, MA, United States). Other chemicals were of analytical grade, and their purity was above 99.5%.

### Preparation of Rhubarb Extracts

Rhubarb was ground to powder and the powder was sieved by 20 meshes. Then rhubarb powder (2 kg) was extracted with 15 L 95% ethanol for 30 min by ultrasonic method for three times. The resulting extract was concentrated under pressure to yield a brown ethanol extract. The ethanol extract obtained was partitioned between water and three organic solvents with different polarities (BU> EA> PE), to yield three new fractions including PE, EA, and BU extracts.

### Animal Model and Treatment With Rhubarb Extracts

Male Sprague-Dawley rats (8 weeks old) were randomized to divide into the following five groups: control group (*n* = 16), CKD group (*n* = 16), CKD+PE group (*n* = 8), CKD+EA group (*n* = 8), and CKD+BU group (*n* = 8). CKD, CKD+PE, CKD+EA, and CKD+BU groups were orally administrated 200 mg/kg body weight of adenine dissolved in 1% (w/v) gum acacia solution once a day continuously for 3 weeks. Control group was similarly provided an equal volume of gum acacia solution. During the adenine gastric gavage periods, after 3 h, CKD+PE, CKD+EA, and CKD+BU groups were administered PE extract (800 mg/kg), EA extract (200 mg/kg), and BU extract (600 mg/kg) by gastric irrigation, respectively, during 6 weeks study periods. The same amount of rhubarb powder was used to yield the PE (800 mg/kg), EA (200 mg/kg), and BU (600 mg/kg) extracts. The administrated dosage of PE, EA, and BU/kg body weight in rats was 20 times the dosage of rhubarb recommended for humans by the Chinese Pharmacopoeia. All the rats were anesthetized with 10% urethane, and blood samples were obtained by carotid artery cannula at week 6. Blood was centrifuged at 13000 rpm for 10 min at 4°C and the supernatant was collected and stored at -80°C for the biochemistry analysis. The left kidney was harvested and immediately washed with physiological saline and stored at -80°C for the following histological and metabolomic study. This study was approved by the Ethical Committee of Northwest University and studies were performed in accordance with the Guide for the Care and Use of Laboratory Animals defined by Ethical Committee of Northwest University.

### Biochemical Analyses

Plasma biochemistry was analyzed as described in detail previously (Zhao et al., 2013c). Biochemical parameters were measured using an Olympus AU640 automatic analyzer.

### Semiquantitation of Renal Phenotypes by Light Microscopic Study

Sections of the paraffin-embedded kidney tissues were cut at a thickness of 2 μm and stained with hematoxylin and eosin reagent. Tubular lesions were characterized by tubular dilation and epithelial desquamation with interstitial expansions. The degree of severity were determined based on the extent of cortical involvement on a scale from 0 to 4: 0, normal cortex; 1, less than 25% of the injured cortex areas; 2, 26 to 50% of the injured cortex areas; 3, 51 to 75% of the injured cortex areas; 4, extensive damage involving more than 75% of the injured cortex areas. Then they were expressed as tubular injury scores (Mizuno et al., 2001). Masson’s trichrome staining was performed as described previously (Zhang et al., 2016b).

### Immunohistochemistry

Immunohistochemical staining of kidney tissues were assessed as described in detail previously (Zhang et al., 2016b). In brief, the paraffin sections were deparafinized, rehydrated, and immersed in 3% hydrogen peroxide to block endogenous peroxidase activity. After blocking, the sections were incubated with antibodies against TGF-β1 (dilution 1: 300; Abcam, United States), TGF-β RII (dilution 1:200; Santa Cruz Biotechnology, Santa Cruz, CA, United States), TGFβRI (dilution 1:100), p-Smad2 (dilution 1:200; Cell Signaling Technology, United States), Smad2 (dilution 1:300; Cell Signaling Technology, United States), p-Smad3 (dilution 1:200; Cell Signaling Technology, United States), Smad3 (dilution 1:100; Cell Signaling Technology, United States), Smad4 (dilution 1:100; Cell Signaling Technology, United States), Smad7 (dilution 1:50; Santa Cruz Biotechnology, United States), collagen I (dilution 1:800; Abcam, United States), fibronectin (dilution 1:500; Abcam, United States), and α-SMA (dilution 1:75; Abcam, United States), then incubated with the horseradish peroxidase-conjugated goat anti-rabbit (ab6721, Abcam, United States) or goat anti-mouse (A21010, Abbkine, United States) secondary antibodies. All histological assays were assessed in ten randomly selected non-overlapping fields at ×400 magnifications. They were photographed and measured by using Image-Pro Plus, version 6.0.

### Western Blotting Analysis

All the Western blotting analyses were performed as described previously (Zhao et al., 2015; Chen et al., 2017a,c; Wang et al., 2017a). Proteins were separated by sodium dodecyl sulfate-polyacrylamide gel electrophoresis gel and transferred to polyvinylidene difluoride (PVDF) membranes (10600023, Amersham^TM^ Hybond^TM^, GE Healthcare, United States) by electroblotting. After blocking in Tween-20 buffer and non-fat milk blocking buffer, the membranes were incubated at 4°C over-night with primary antibodies against proteins including E-cadherin (1:500, Abcam, United States), FSP1 (1:1000, Abcam, United States), vimentin (1:200, Abcam, United States), and collagen III (1:5000, Abcam, United States). Membranes were washed and incubated with appropriate horseradish peroxidase-conjugated goat anti-mouse (1:5000, Abbkine, United States) and goat anti-rabbit (1:5000, Abcam, United States) secondary antibodies for 1 h. Blots were detected by enhanced chemiluminescence (RPN2232, GE Healthcare, United States) and band density was quantified by Image J 1.48v software. GAPDH served as the internal control.

### Sample Preparation and UPLC-MS Analysis

Kidney samples were prepared as described previously (Zhao et al., 2013a). In brief, kidney samples (100 mg) were homogenized in 0.5 mL of acetonitrile in an ice bath. Samples were then vortexed for 2 min, and put on ice in between. Following centrifugation (13,000 rpm, 10 min, 4°C), 300 μL of supernatant was removed and then lyophilized. Before analysis, the extract was resuspended in 100 μL acetonitrile/water (4:1) for UPLC analysis. The UPLC analysis was performed on a 2.1 mm × 100 mm ACQUITY 1.8 μm HSS T3 using a Waters Acquity^TM^ UPLC system equipped with a Waters Xevo^TM^ G2 QT of MS. The mobile phase consisted of water (A) and acetonitrile (B), each containing 0.1% formic acid. The optimized UPLC elution conditions were: 0-2.0 min, 1-60% B; 2.0-6.0 min, 60-85% B; 6.0-8.0 min, 85-99% B; and 8.0-10.0 min, 99.0-1.0% B. The flow rate was 0.45 mL/min. Autosampler was maintained at 4°C. One microliter of sample solution was injected for each run.

Mass spectrometry (MS) of the optimal conditions were as follows: capillary voltage: 2.5 kV, cone voltage: 30 V, desolvation gas temperature: 500°C, source temperature: 120°C, desolvation gas flow: 600 L/h, cone gas flow: 50 L/h, The scan range was from 50 to 1200 m/z. Leucine enkephalin was used for accurate mass acquisition. Waters MassLynx v4.1 was used for all the acquisition and analysis of data in both positive ion mode and negative ion mode.

### Data Analysis

The acquired raw data from UPLC-HDMS analysis in positive and negative ion modes were first pre-processed by Markerlynx XS and Progenesis QI (Waters, Manchester, United Kingdom). The spectral data were conducted by PCA to visualize general clustering, trends, or outliers among the observations. OPLS-DA and PLS-DA was utilized to validate the PCA model and identify the differential metabolites. The variables were selected based on variable importance in the projection (VIP > 1.0) from the normalized peak intensity. Variables were further selected by Student’s *t*-test of with a threshold of *p* < 0.05 in SPSS 19.0. The resulting *p*-values from Student’s *t*-test were further adjusted by a false discovery rate (FDR) based on the Benjamini–Hochberg method. Fold changes from each group/control group or each group/CKD group and ROC curve was performed by MetaboAnalyst 3.0. Significant variables were identified and confirmed by comparing MS data, MS/MS fragments, molecular weights, and elemental compositions with the available reference chemicals or with available biochemical databases, such as HMDB^1^, ChemSpider^2^, and KEGG^3^.

## Results

### Biochemical Parameters

**FIGURE 1:**
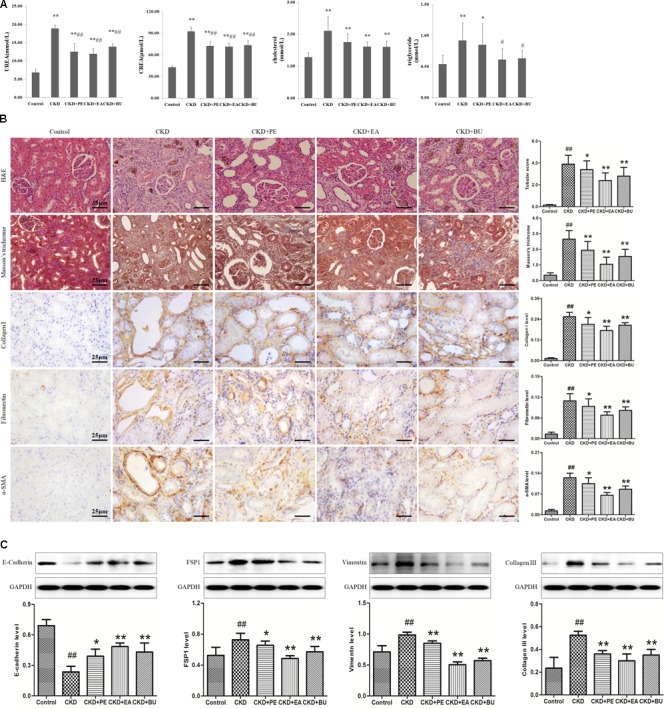
**(A)** Biochemical parameters including cholesterol, triglyceride, creatinine (CREA), urea in the normal control, CKD+PE, CKD+EA, and CKD+BU groups. **(B)** Representative photomicrographs of the H&E staining, Masson’s trichrome staining, collagen I, Fibronectin, and α-SMA immunohistochemistry from kidney sections in control, CKD, CKD+ PE, CKD+ EA, and CKD+ BU groups. **(C)** Renal fibrotic protein expression in the five different groups. Expression levels of E-cadherin, FSP1, vimentin, and collagen III proteins were determined in control, CKD, CKD+ PE, CKD+ EA, and CKD+ BU groups by Western blotting. Bar graphs depicted expression intensity of E-cadherin, FSP1, vimentin, and collagen III proteins. GAPDH served as the loading control. ^##^*p* < 0.01 compared to controls; ^∗^*p* < 0.05, ^∗∗^*p* < 0.01 compared to CKD rat.

As shown in **Figure 1A**, CKD rats had significantly higher cholesterol, triglyceride, creatinine (CREA), and urea in relative to control rats. Treatment with PE, EA, and BU groups could lower the cholesterol, triglyceride CREA, and urea to some degree. Especially, EA-treated (EA+CKD) and BU-treated (BU+CKD) groups showed a significant decrease in serum triglyceride, CREA and urea concentrations in relative to CKD group. PE-treated (PE+CKD) group showed a significant decrease in serum CREA and urea concentrations in relative to CKD group.

### Histological Data and Western Blotting

Representative photomicrographs of the hematoxylin and eosin (H&E) and Masson’s trichrome stained rats’ kidney tissues are shown in **Figure 1B**. Kidney tissues from the CKD rats showed severe tubular dilatation, tubular atrophy, and widened interstitial space with severe inflammatory cell infiltration. The administration of rhubarb extracts significantly attenuated the tubulointerstitial damage (**Figure 1B**). Myofibroblasts characterized by the expression of α-SMA play important roles in development of fibrotic lesions. α-SMA was extensively expressed in renal tubules of the rats with CKD. Apart from α-SMA, other renal fibrotic proteins including fibronectin and collagen I had a high expression in CKD rats. Expressions of all of the above proteins were significantly reduced by treatment with PE, EA, and BU extracts (**Figure 1B**). Western blotting analyses of the kidney tissue extracts also revealed that the same result (**Figure 1C**). Collagen III was extensively expressed in CKD rats and significantly reduced in treatment groups. There was a clearly reduced expression of E-cadherin and increase in expression of the mesenchymal markers vimentin in the CKD group, while expression of E-cadherin was increased and expression of vimentin was decreased in treatment groups. FSP1, a fibroblast marker, was extensively expressed in CKD rats and significantly reduced in treatment groups.

### TGF-β/Smad Pathway

**FIGURE 2:**
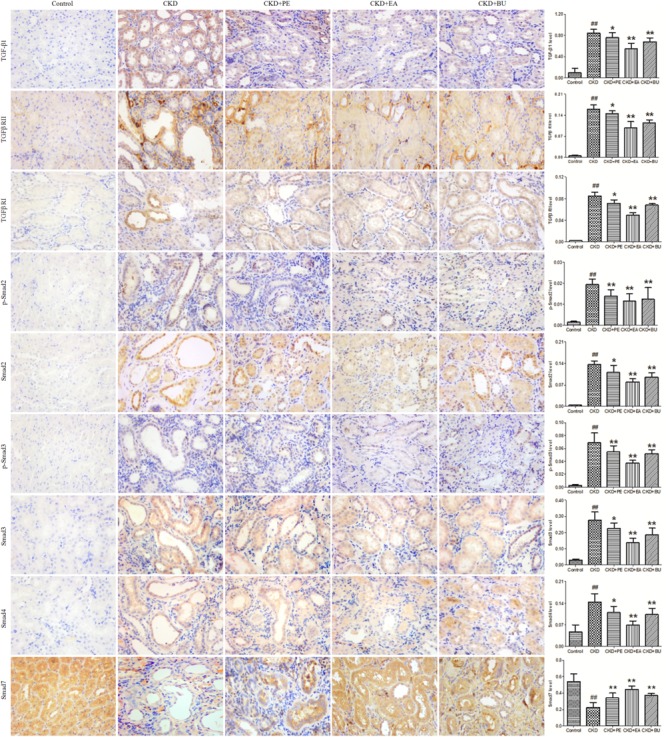
Representative photomicrographs of the TGF-β1, TGF-β RI, TGF-β RII, Smad2, p-Smad2, Smad3, p-Smad3, Smad4, and Smad7 immunohistochemistry from kidney sections in normal control rat, CKD rat, CKD+PE rat, CKD+EA rat, and CKD+BU rat. ^##^*p* < 0.01 compared to controls; ^∗^*p* < 0.05, ^∗∗^*p* < 0.01 compared to CKD rat.

Expressions of proteins in TGF-β/Smad pathway in the kidney tissues are shown in **Figure 2**. Kidney tissues obtained from the CKD group showed a significant up-regulation in TGF-β1, TGF-βRI, TGF-βRII, Smad2, p-Smad2, Smad3, p-Smad3, and Smad4 while it showed a significant down-regulation in Smad7, pointing to activation of TGF-β/Smad signaling pathway. Treatments with PE, EA, and BU could markedly attenuate the up-regulation in TGF-β1, TGF-β RI, TGF-β RII, Smad2, p-Smad2, Smad3, p-Smad3, and Smad4 and enhance the down-regulation in Smad7, indicating that rhubarb extracts exert nephroprotective effect by rebalancing the TGF-β/Smad signaling pathway.

### Metabolic Profile and Multivariate Analysis

**FIGURE 3:**
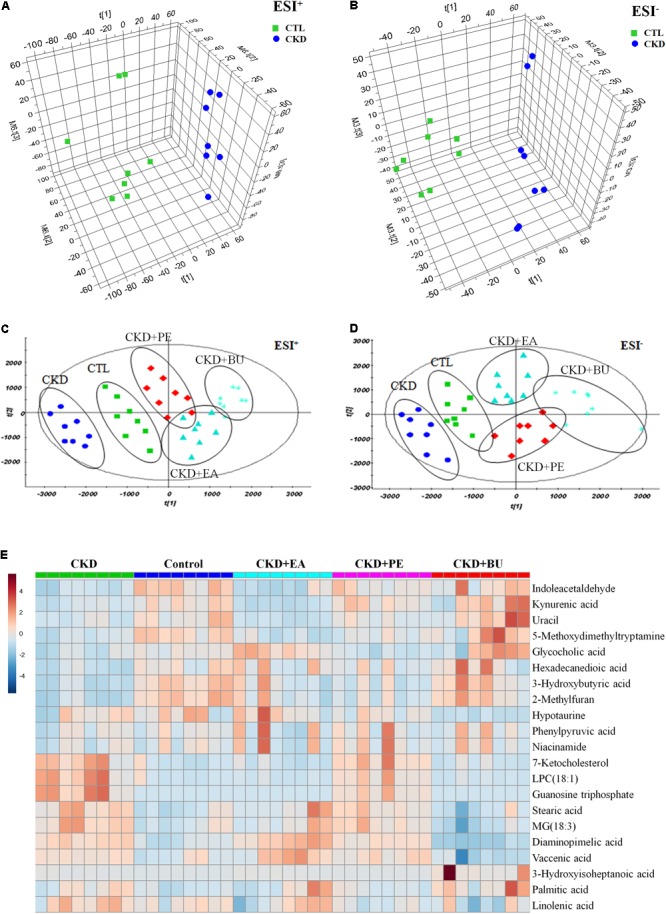
Metabolomic profiling of tissue samples. **(A)** PCA scores plot from CKD samples and control samples in positive ion mode. **(B)** PCA scores plot from CKD samples and control samples in negative ion mode. **(C)** PCA scores plot of comparing control, CKD, CKD+PE, CKD+EA, and CKD+BU groups in positive ion mode. **(D)** PCA scores plot of comparing control, CKD, CKD+PE, CKD+EA, and CKD+BU groups in positive ion mode. **(E)** Heat maps for 21 altered metabolites. Red and blue indicate increased and decreased levels, respectively. Rows, metabolites; columns, tissue samples.

Metabolic profiling of tissue samples was acquired using UPLC-QTOF/HDMS in the positive and negative ESI modes. The representative total ion chromatograms of all groups are presented in **Supplementary Figure S1**. To evaluate the alterations of metabolome in CKD rats, an unsupervised PCA was performed using data from CKD group and control group. PCA score plots could readily be divided into two clusters (**Figures 3A,B**), indicating that plasma metabolic states of CKD group were significantly changed in relative to control group. On the other hand, CKD+PE, CKD+EA, and CKD+BU affected the metabolite variations in different directions and PCA analysis showed a clear separation between these three groups and the untreated CKD group (**Figures 3C,D**). PLS-DA was employed to select the differential metabolites from control and CKD groups. Initially, 191 and 150 variables with PLS-DA based on VIP values greater than 1.0 in positive ion and negative ion modes were selected. Among them, 22 and 23 metabolites were identified according to our previously reported methods (Zhao et al., 2012a,b). Subsequently, these 45 metabolites were further selected using Student’s *t*-test (*p* < 0.05) with a FDR < 0.05. Finally 40 differentially expressed metabolites were selected and summarized in **Table 1**.

**Table 1:** Differential metabolites from different groups at weeks 6.

Primary ID	CKD vs. Control						CKD+PE vs. CKD		CKD+EA vs. CKD		CKD+BU vs. CKD	
	ESI	Vip^a^	FC^b^	*t*-Test^c^	FDR^d^	AUC	FC^b^	*t*-Test^c^	FC^b^	*t*-Test^c^	FC^b^	*t*-Test^c^
3-Hydroxybutyric acid	+	2.23	0.55	9.28E-09	6.50E-08	0.99	1.21	1.56E-01	1.19	2.07E-01	1.55	1.04E-04
L-valine	+	2.23	1.98	1.81E-09	3.81E-08	1.00	0.96	5.29E-01	1.19	6.28E-02	1.39	2.83E-04
Beta-citryl-L-glutamic acid	+	2.20	0.76	1.86E-06	3.00E-06	0.92	0.85	3.29E-04	0.79	2.14E-05	0.81	6.68E-05
2-Methylfuran	+	2.17	0.53	2.86E-08	8.57E-08	1.00	1.20	2.16E-01	1.13	4.29E-01	1.51	2.82E-04
PE(16:0/P-16:0)	+	2.17	1.57	2.36E-08	8.26E-08	0.96	1.10	3.90E-02	1.20	1.05E-03	1.25	4.33E-07
LPC(18:1)	+	2.16	11.33	7.65E-08	1.46E-07	0.98	0.78	2.36E-01	0.08	4.34E-08	0.07	2.08E-08
PC(36:5)	+	2.16	1.72	1.34E-08	7.03E-08	1.00	1.06	3.04E-01	1.23	2.31E-02	1.79	1.22E-06
Octacosanoic acid	+	2.15	1.50	9.19E-09	9.65E-08	0.98	1.03	5.40E-01	1.20	6.44E-03	1.16	1.66E-03
Guanosine triphosphate	+	2.14	13.83	1.90E-08	7.98E-08	1.00	0.68	6.77E-02	0.07	1.90E-08	0.14	1.90E-08
7-Ketocholesterol	+	2.14	4.55	3.75E-07	6.56E-07	0.97	1.27	2.07E-01	0.22	6.75E-07	0.26	4.23E-07
PC(20:2/22:5)	+	2.11	5.28	3.62E-08	8.45E-08	0.97	1.27	4.10E-02	1.28	8.44E-02	1.47	2.92E-04
Docosahexaenoic acid	+	2.11	2.56	2.87E-08	7.53E-08	0.97	1.55	5.25E-08	1.49	2.29E-04	2.29	1.84E-05
5-Hydroxy-6-methoxyindole glucuronide	+	2.08	0.39	5.61E-08	1.18E-07	0.95	1.72	5.73E-02	1.47	2.11E-01	1.08	7.56E-01
Hexadecenal	+	2.06	0.88	3.36E-03	4.15E-03	0.91	0.82	5.35E-07	0.76	2.01E-08	0.74	1.18E-10
Cervonoyl ethanolamide	+	2.06	2.36	2.35E-06	3.52E-06	0.90	1.09	3.40E-01	1.03	7.90E-01	1.70	2.59E-04
Hexadecanedioic acid	+	1.55	0.57	3.08E-04	4.32E-04	0.85	1.80	1.31E-02	1.52	4.79E-02	1.68	2.50E-02
Kynurenic acid	+	1.45	0.75	1.81E-03	2.37E-03	0.81	1.51	2.43E-05	0.63	3.20E-05	1.48	2.00E-02
Phenylpyruvic acid	+	1.34	0.89	1.09E-02	1.27E-02	0.71	1.39	1.35E-02	1.11	1.93E-01	1.05	4.10E-01
Niacinamide	+	1.24	0.87	2.83E-02	2.83E-02	0.70	1.50	8.92E-03	1.15	1.25E-01	1.07	3.13E-01
Uracil	+	1.15	0.71	1.11E-02	1.23E-02	0.78	1.19	1.10E-01	0.73	2.74E-02	1.79	4.16E-03
Hypotaurine	+	1.13	0.66	2.50E-02	2.62E-02	0.72	1.42	7.07E-02	1.92	7.25E-04	0.88	5.12E-01
Uric acid	+	1.38	1.60	1.82E-04	2.67E-04	0.87	1.24	6.02E-02	2.12	2.41E-05	3.15	7.95E-12
Stearic acid	–	2.12	1.83	4.88E-08	2.20E-07	0.96	0.86	2.39E-02	0.84	1.59E-01	0.62	7.27E-04
MG(18:3)	–	2.03	1.65	1.52E-07	5.47E-07	0.96	0.87	2.37E-02	0.75	2.85E-03	0.53	5.72E-07
Diaminopimelic acid	–	1.97	1.72	1.63E-08	2.93E-07	0.98	0.86	1.56E-03	1.12	1.90E-01	0.34	4.83E-06
Uridine	–	1.97	1.44	4.75E-08	2.85E-07	1.00	1.14	1.84E-02	1.00	9.56E-01	1.24	6.51E-02
5-Methoxydimethyltryptamine	–	1.93	0.37	2.07E-08	1.86E-07	0.95	1.80	5.57E-03	0.72	2.24E-01	2.48	3.51E-03
Palmitic acid	–	1.89	1.49	3.11E-05	4.31E-05	0.89	0.74	2.55E-04	0.90	4.96E-01	0.91	4.00E-01
Phenyllactic acid	–	1.89	0.73	4.02E-06	8.04E-06	0.92	1.10	2.25E-01	0.73	1.61E-04	0.91	3.23E-01
Adrenic acid	–	1.83	1.89	9.10E-05	1.09E-04	0.84	1.21	8.82E-02	1.11	6.33E-01	1.43	2.98E-02
Glycocholic acid	–	1.79	0.48	8.72E-06	1.31E-05	0.95	0.88	4.86E-01	2.46	3.19E-06	2.55	2.26E-03
Linolenic acid	–	1.79	1.27	8.30E-05	1.07E-04	0.89	0.84	4.50E-04	0.82	2.00E-02	0.78	3.49E-04
MG(20:0)	–	1.76	0.26	2.36E-06	6.07E-06	1.00	0.17	4.55E-07	0.08	3.06E-08	0.03	6.21E-09
Fumaric acid	–	1.73	1.27	5.75E-06	1.04E-05	0.92	1.05	1.98E-01	1.07	1.33E-01	0.94	4.54E-01
Indoleacetaldehyde	–	1.73	0.40	1.93E-06	5.80E-06	0.93	1.88	8.89E-04	0.78	4.18E-01	2.13	2.69E-03
3-Hydroxyisoheptanoic acid	–	1.72	1.76	3.27E-06	7.36E-06	0.94	0.77	2.84E-03	0.94	4.36E-01	4.32	2.92E-02
Vaccenic acid	–	1.70	1.30	6.41E-06	1.05E-05	0.92	1.06	2.10E-01	1.20	3.69E-03	0.76	9.07E-03
Arachidonic acid	–	1.63	1.52	6.97E-04	7.84E-04	0.84	1.14	1.65E-01	0.91	4.25E-01	1.20	1.14E-01
Docosapentaenoic acid	–	1.35	1.48	2.27E-02	2.27E-02	0.72	0.92	5.09E-01	1.21	3.92E-01	1.17	4.05E-01
Linoleic acid	–	1.29	1.52	2.06E-02	2.18E-02	0.73	0.89	4.12E-01	1.13	5.78E-01	1.04	8.26E-01

*^*a*^VIP was obtained from PLS-DA model with a threshold of 1.0. ^*b*^FC was obtained by comparing CKD group vs. control group or each group vs. CKD group; FC with a value >1 indicated a relatively higher intensity, whereas a value <1 indicated a relatively lower intensity. ^*c*^p-Values from Student’s *t*-test. ^*d*^Value of FDR was obtained from the adjusted *p*-value of FDR correction by Benjamini–Hochberg method.*

**FIGURE 4:**
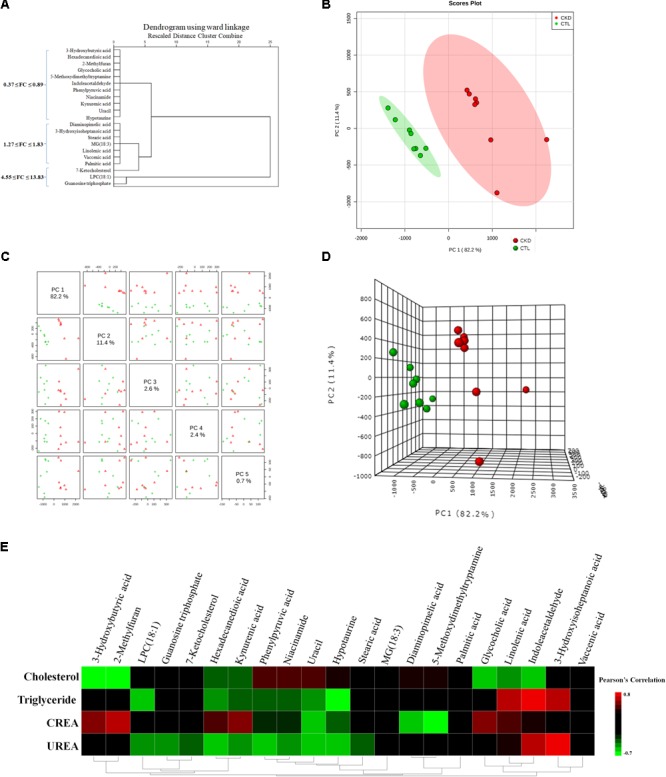
**(A)** Hierarchical clustering dendrogram of the identified significant metabolites. The **(B)** 2D PCA and **(D)** 3D PCA score scatter plot using 21 differential metabolites from 8 CKD rats and 8 control rats. The unsupervised PCA score plots showed that 21 differential metabolites could separate CKD group from the control group. **(C)** Different principal components have a different contribution to separating CKD from healthy controls in this study. Red triangles and green crosses represent CKD samples and control samples, respectively. **(E)** Associations of metabolites with clinical indices. Heat map of the Pearson’s rank correlation coefficient between 4 clinical indices and 21 differential metabolites.

Based on the data presented in **Table 1**, we found out that treatment with EA, BU, and PE extracts exerted some therapeutic effects by normalizing or partially reversing the CKD-induced alterations in the 21 biomarker metabolites. This result is consistent with the observed improvements in serum biochemical parameters and findings. Then a heatmap was performed to visualize the relative levels of the 21 differential metabolites in each group (**Figure 3E**). The relationships of the differential metabolites were analyzed by hierarchical clustering analyses (**Figure 4A**). These metabolites were clustered into three groups in the light of their fold change (CKD/Control). Another independent cohort of tissue samples (8 controls and 8 CKD rats) was analyzed to validate the reliability of these 21 differential metabolites. The results confirmed that the 21 metabolites could separate CKD rats from the controls (**Figures 4B–D**).

**FIGURE 5:**
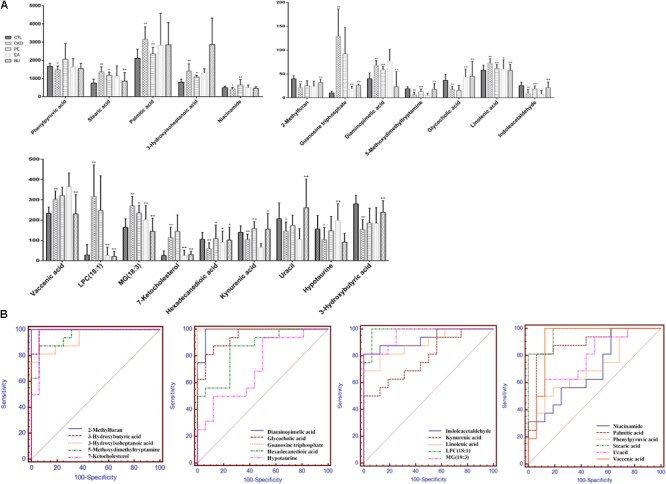
Relative concentrations of the 21 metabolites in control, CKD, CKD+PE, CKD+EA, and CKD+BU groups **(A)** and their receiver operating characteristic curves **(B)**. ^∗^*p* < 0.05, ^∗∗^*p* < 0.01 compared to control group; ^+^*p* < 0.05, ^++^*p* < 0.01 compared to CKD group.

To investigate the association of significant alterations in metabolome with severity of CKD, we performed Pearson rank correlation to directly measure the correlations between 4 clinical indices and the 21 differential metabolites (**Figure 4E**). The analysis showed that 3-hydroxybutyric acid, 2-methylfuran, glycocholic acid, linolenic acid and indoleacetaldehyde were negatively correlated while phenylpyruvic acid, niacinamide, and uracil were positively correlated with cholesterol. LPC(18:1), hexadecanedioic acid, kynurenic acid, phenylpyruvic acid, niacinamide, uracil, and hypotaurine were negatively correlated while linolenic acid, indoleacetaldehyde and 3-hydroxyisoheptanoic acid were positively correlated with triglyceride. 3-hydroxybutyric acid, 2-methylfuran, hexadecanedioic acid, kynurenic acid, glycocholic acid and linolenic acid were positively correlated while uracil, hypotaurine, diaminopimelic acid and 5-methoxydimethyltryptamine were negatively correlated with creatinine. LPC(18:1), guanosine triphosphate, 7-ketocholesterol, hexadecanedioic acid, kynurenic acid, phenylpyruvic acid, niacinamide, uracil, and hypotaurine were negatively correlated while indoleacetaldehyde and 3-hydroxyisoheptanoic acid were positively correlated with urea. **Figure 5** showed the intensities of 21 individual metabolites and their receiver operating characteristic curve (ROC).

## Discussion

Adenine-induced CKD model characterized by severe tubulointerstitial nephropathy has been widely used to study CKD (Zhao et al., 2012a,d, 2014b). Long-term feeding of adenine to rats produced metabolic abnormalities resembling chronic renal insufficiency in humans (Yokozawa et al., 1986). This result has been confirmed by biochemical and histological findings (Zhang et al., 2015). TGF-β/Smad signaling pathway plays a key role in renal fibrosis, which has been widely accepted (Meng et al., 2013, 2015). TGF-β1 binds TGF-β RII to activate TGF-β RI and phosphorylates the downstream receptor-associated R-Smads, including Smad2 and Smad3 (Wrana et al., 1994). Then the p-Smad2 and p-Smad3 form a heteromeric complex with a common Smad, Smad4, and translocates into the nucleus to induce the transcription of target genes in collaboration with various co-activators and co-repressors. Smad4 is a common Smad for TGF-β/BMP signaling and plays a critical role for shuttling Smad2/3 and Smad1/5/8 into the nucleus (Chen et al., 2018b). Of note is that Smad7, an inhibitory Smad, can be induced by TGF-β1 to block the over activation of TGF-β signals via inhibition of TGF-β RI and Smad2/3 (Chen et al., 2018a,b). TGF-β1 not only induces Smad7 transcription, but also promotes the degradation of Smad7 by activating the Smad3-dependent Smurfs/arkadia-mediated ubiquitin–proteasome degradation pathway (Chen et al., 2018b). In CKD group, due to the high level of active TGF-β1, the level of Smad7 is significantly decreased. In **Figure 2**, the expression of TGF-β1 and its receptors increased in CKD rats. The level of downstream proteins such as Smad2, Smad3, p-Smad2, p-Smad3, and Smad4 increased in CKD rats too. In addition, the expression of Smad7 was down-regulated in CKD rats. These results indicated the activation of TGF-β/Smad signaling axis. Up-regulation of TGF-β1, TGF-β RI, TGF-β RII, Smad2, p-Smad2, Smad3, p-Smad3, and Smad4 can be significantly down-regulated by the treatments with PE, EA, and BU. Down-regulation of Smad7 can be significantly up-regulated by the treatments with PE, EA, and BU. These data indicated that rhubarb extracts protected rats from the development of renal fibrosis by rebalancing the TGF-β/Smad signaling pathway. Thus, targeting TGF-β/Smad signaling may represent a specific and effective therapy for CKD associated with renal fibrosis.

**FIGURE 6:**
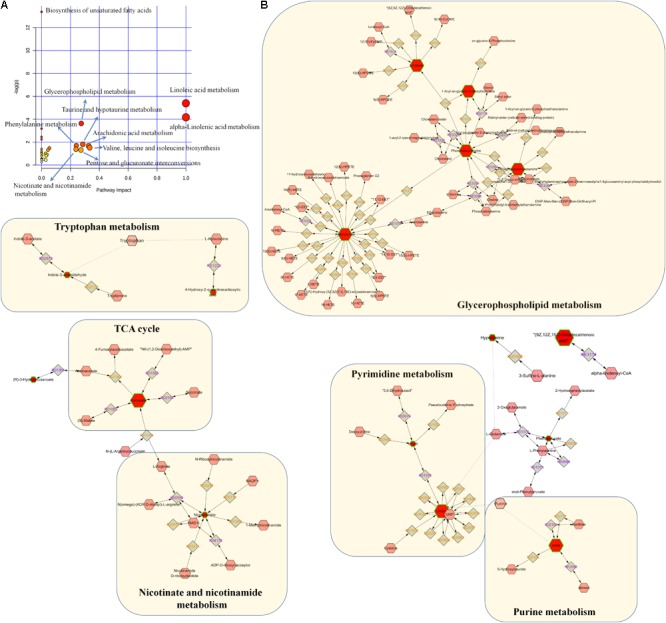
Pathway analysis of the differential metabolites. **(A)** Pathway analysis of 40 differential metabolites. The size and color of each circle was based on pathway impact value and *p*-value, respectively. **(B)** Metabolic pathways were visualized by means of cytoscape software. The differential metabolites in our study were represented by red hexagons. Hexagons with green lines mean that the alteration of the biomarkers in CKD had statistical significance. The size of hexagons indicated the FC of the corresponding metabolite in CKD in relative to controls. In addition, pink hexagons indicated metabolites participating in the metabolic pathway but not been detected in our study. Diamonds mean that the metabolites convert to the products by the reactions.

On the basis of our metabolomic findings, 191 and 150 altered metabolites were found in positive ion and negative ion modes. After the comprehensive screening and validation workflow, 40 differential metabolites were eventually identified which contribute to differentiating CKD rats from controls and they reflected dysregulation of the metabolic pathways including tryptophan metabolism, taurine and hypotaurine metabolism, purine metabolism, pyrimidine metabolism, fatty acid metabolism, glycerophospholipid metabolism, nicotinate and nicotinamide metabolism, and TCA cycle (**Figure 6**). The 21 abnormal altered metabolites could be normalized or partially reversed by the treatment with EA, BU, or PE extracts. Four out of 21 metabolites including hexadecanedioic acid, kynurenic acid, MG(18:3), and linolenic acid were improved in all three rhubarb extract groups. 3-hydroxybutyric acid, 2-methylfuran, uracil and vaccenic acid were improved only in BU group. Hypotaurine was improved only in EA group. Phenylpyruvic acid, niacinamide and palmitic acid were improved only in PE group.

Glycerophospholipids are the fundamental components of the cell membranes. Apart from their function in cell membranes, they play an important role in other cellular processes such as signal induction and transport. Glycerophospholipids metabolize to lysophosphatidic acid (LPA) by the addition or transfer of the fatty acid chains to the glycerol backbone. LPA convert to the next intermediate phosphatidic acid (PA) by acylation. PA can be dephosphorylated leading to the formation of diacylglycerol which is essential in the synthesis of phosphatidylcholine (PC) (Ecker and Liebisch, 2014). PC can then be further converted to other species of glcerophospholipids such as phosphatidylserine (PS) and phosphatidylethanolamine (PE) (Ecker and Liebisch, 2014). A growing number of studies have indicated that abnormal glycerophospholipids metabolism contributes to the progression of renal disease in patients and animals models. Increase in LPC(18:1), PC(20:2/22:5), PC(36:5), PE(16:0/P-16:0), MG(18:3), and decrease in MG(20:0) were observed in rats with CKD. Significantly increased PC and PE is in agreement with a previous report on the levels of PC and PE in POKO mice which exhibit rapid progression of renal disease (Martinez-Garcia et al., 2012) and in adenine-induced CKD rats (Zhang et al., 2016b). Increased LPC (18:1) can be reversed to normal level in CKD rats by the treatments with EA and BU extracts. Increased MG (18:3) can be reversed to normal level in CKD rats by the treatments with PE, EA, and BU extracts.

Fatty acids are precursors of various biologically important molecules such as triglycerides, phospholipids, second messengers, local hormones, and ketone bodies (Berg et al., 2002). Fatty acids, an important source of energy for animal, can be completely oxidized to CO_2_ and water by β-oxidation and the citric acid cycle. This process yields the most ATP on an energy per gram basis compared to the other common nutrients including carbohydrates and protein. Accumulation of FFA and SFA are strongly related to higher risk of cardiovascular disease in patients with kidney disease (Friedman et al., 2013). It has been reported that the serum levels of free fatty acids (FFA) and SFA are significantly increased in patients with kidney disease (Chen et al., 2017b). Wang et al. (2016) reported that FFA and SFA increased significantly in prehemodialysis patients in relative to the healthy subjects and they decreased significantly after hemodialysis. In addition, Kang et al. (2015) found that humans and mice with tubulointerstitial fibrosis had lower expression of key enzymes and regulators of fatty acid oxidation and increased intracellular lipid deposition. Moreover, the level of a number of FFA including docosahexaenoic acid, stearic acid, palmitic acid, linolenic acid, AA increased in CD36 transgenic mice of kidney fibrosis in relative to controls (Kang et al., 2015). In our study, docosahexaenoic acid, stearic acid, palmitic acid, linolenic acid, AA increased significantly in CKD rats compared to controls, which is consistent with abovementioned report. Besides, increased level of some other FFA, for instance octacosanoic acid, adrenic acid, docosapentaenoic acid, and linoleic acid were also observed in the CKD rats compared to controls. Restoration of fatty acid oxidation by genetic or pharmacological methods could effectively protect mice from the development of tubulointerstitial fibrosis (Kang et al., 2015). Our results showed that treatments with PE, EA, or BU extracts reversed the rise in stearic acid, palmitic acid, and linolenic acid to normal level in CKD rats, indicating that rhubarb extracts exert renoprotective effect by partially improving the fatty acid metabolism.

Hypotaurine is a product of enzyme cysteamine dioxygenase in taurine and hypotaurine metabolism pathway. It function as an antioxidant and a protective agent (Poljšak and Fink, 2014). Previous study found markedly alterations in oxidative stress in early stage CKD. In particular, the free-radical scavengers hypotaurine were decreased in the urine of patients, but conversely hypotaurine and taurine were increased in the serum (Hayashi et al., 2011). Low level of the hypotaurine was observed in the tissue samples of CKD rats compared to the controls. Treatment with the EA extract reversed the increase in hypotaurine to normal levels in CKD rats. Additionally, we found decreased hypotaurine were negative correlated with triglyceride and urea in CKD rats. Uric acid is the final oxidation product of purine metabolism (Yamamoto et al., 2005). Abnormality of the uric acid concentration is sensitive indicator of certain pathological states, including gout, hyperuricemia, renal failure, toxemia during pregnancy (Dutt et al., 2003). Previous study indicated plasma uric acid increased significantly in patients with ESRD and fell after hemodialysis (Shahbazian et al., 2009). High level of uric acid in blood causes deposition of urate crystals, which could ultimately lead to chronic joint inflammation and kidney damage. In our study, uric acid showed a significant increase in CKD rats which is in agreement with previous reports.

## Conclusion

Adenine-induced chronic tubulointerstitial fibrosis showed the activation of TGF-β/Smad signaling axis as well as marked abnormalities in tryptophan metabolism, taurine and hypotaurine metabolism, purine metabolism, pyrimidine metabolism, fatty acid metabolism, glycerophospholipid metabolism, nicotinate and nicotinamide metabolism, and TCA cycle. Treatments with PE, EA, and BU extracts of rhubarb attenuated kidney injury, improved renal function, partially reversed abnormalities of tissue metabolome and suppressed renal fibrosis by rebalancing the TGF-β/Smad signaling pathway.

## Author Contributions

Z-HZ, S-CM, and Y-YZ designed the experiments. M-HL, DL, HC, and Y-YZ collected and analyzed the data. Z-HZ and Y-YZ wrote the manuscript. N-HT and D-QC revised the paper. All authors read and approved the paper.

## Conflict of Interest Statement

The authors declare that the research was conducted in the absence of any commercial or financial relationships that could be construed as a potential conflict of interest.

## Footnotes

1 http://www.hmdb.ca/

2 http://www.chemspider.com/

3 www.kegg.jp

## Abbreviations

α-SMA: alpha smooth muscle actin
BU: *n*-butanol extract
CKD: chronic kidney disease
EA: ethyl acetate extract
ECM: extracellular matrix
FFA: free fatty acids
FSP1: fibroblast-specific protein 1
GAPDH: glyceraldehyde-3-phosphate dehydrogenase
PCA: principal component analysis
PE: petroleum ether extract
PLS-DA: partial least squares-discriminant analysis
SFA: saturated fatty acids
TGF-β RI: transforming growth factor-β receptor I
TGF-β RII: transforming growth factor-β receptor II
TGF-β: transforming growth factor-β
VIP: variable importance in the projection
