# Supplementary material for: Rhubarb Protect Against Tubulointerstitial Fibrosis by Inhibiting TGF-β/Smad Pathway and Improving Abnormal Metabolome in Chronic Kidney Disease
Source: Front Pharmacol. 2018 Sep 13;9:1029. doi: 10.3389/fphar.2018.01029 (PMC6146043; doi:10.3389/fphar.2018.01029)
Supplement: Supplementary file 1 [file Table_1.DOC]

*Supporting Information*

Rhubarb protect against tubulointerstitial fibrosis by inhibiting TGF-β/Smad pathway and improving abnormal metabolome in chronic kidney disease

Zhi-Hao Zhang^1,2,#^, Ming-Hua Li^3,#^, Dan Liu^1,#^, Hua Chen^1^, Dan-Qian Chen^1^, Ning-Hua Tan^2^, Shuang-Cheng Ma^3,*^, Ying-Yong Zhao^1,*^

^1^ Key Laboratory of Resource Biology and Biotechnology in Western China, Ministry of Education, School of Life Sciences, Northwest University, No. 229 Taibai North Road, Xi’an, Shaanxi 710069, China

^2^ School of Traditional Chinese Pharmacy and State Key Laboratory of Natural Medicines, China Pharmaceutical University, Nanjing 211198, China

^3^ National Institutes for Food and Drug Control, State Food and Drug Administration, No. 2 Tiantan Xili, Beijing 100050, China

^#^ Zhi-Hao Zhang, Ming-Hua Li and Dan Liu are the co-first authors.

* Corresponding authors:

Shuang-Cheng Ma, PhD, Professor

National Institutes for Food and Drug Control, State Food and Drug Administration, Beijing 100050, China, Tel: +86 10 67095272; Fax: +86 10 67095887; E-mail: masc@nifdc.org.cn (S.C. Ma)

Ying-Yong Zhao, PhD, MD, Professor

School of Life Sciences, Northwest University, Xi’an, 710069, China, Tel: +86 29 88305273; Fax: +86 29 88303572; E-mail: zyy@nwu.edu.cn; zhaoyybr@163.com

Figure S1

Figure S1. The representative BPI chromatograms (ESI^+^) of metabolomic samples (from top to bottom are control, CKD, CKD+PE, CKD+EA and CKD+BU groups respectively)
